# Cortical excitability and multifidus activation responses to transcranial direct current stimulation in patients with chronic low back pain during remission

**DOI:** 10.1038/s41598-023-43597-7

**Published:** 2023-09-27

**Authors:** Peemongkon Wattananon, Khin Win Thu, Soniya Maharjan, Kanphajee Sornkaew, Hsing-Kuo Wang

**Affiliations:** 1 Spine Biomechanics Laboratory, Faculty of Physical Therapy, Mahidol University, 999 Phuttamonthon 4 Road, Salaya, 73170 Nakhon Pathom Thailand; 2 Department of Physical Therapy, Faculty of Allied Health Sciences, Naresuan University, 99 Nakhonsawan-Phitsanulok Road, Tumbon Thapho, Phitsanulok, 65000 Thailand; 3 Sports Physiotherapy Lab, School and Graduate Institute of Physical Therapy, College of Medicine, National Taiwan University, No.17, Xuzhou Rd., Zhongzheng District, Taipei City 100, Taiwan

**Keywords:** Rehabilitation, Rehabilitation

## Abstract

Evidence indicates that patients with chronic low back pain (CLBP) have lumbar multifidus muscle (LM) activation deficit which might be caused by changes in cortical excitability. Anodal transcranial direct current stimulation (a-tDCS) can be used to restore cortical excitability. This study aimed to (1) determine the immediate effects of a-tDCS on the cortical excitability and LM activation and (2) explore the relationship between cortical excitability and LM activation. Thirteen participants with CLBP during remission and 11 healthy participants were recruited. Cortical excitability (peak-to-peak motor evoked potential amplitude; P2P and cortical silent period; CSP) and LM activation were measured at pre- and post-intervention. We found significant difference (*P* < 0.05) in P2P between groups. However, no significant differences (P > 0.05) in P2P, CSP and LM activation were found between pre- and post-intervention in CLBP. The CLBP group demonstrated significant correlation (*P* = 0.05) between P2P and LM activation. Although our finding demonstrates change in P2P in the CLBP group, one-session of a-tDCS cannot induce changes in cortical excitability and LM activation. However, moderate to strong correlation between P2P and LM activation suggests the involvement of cortical level in LM activation deficit. Therefore, non-significant changes could have been due to inadequate dose of a-tDCS.

## Introduction

Low back pain (LBP) is a common musculoskeletal problem affecting nearly 60 to 80% of the population throughout their life^1^. It can occur at any age from adolescents to the elderly and causes individual and social problems^1^. Once an individual suffers from LBP, a high recurrence rate occurs throughout their lifetime, approximately 60 and 70% yearly^2^. If left untreated, it might turn into a chronic stage with a more complex mechanism difficult to be treated^3–6^. In addition, any intervention that does not address the underlying mechanism could be responsible for a high recurrence rate^3–6^. Therefore, the intervention should be designed and focus on a specific underlying mechanism.

Movement control impairment (MCI) has been proposed as a mechanism underlying chronic low back pain (CLBP)^4–8^. MCI is defined as poor control and coordination of the lumbar and pelvic segments during movements^6^. One recent study demonstrated greater positive movement control battery tests in patients with CLBP suggesting that CLBP could be caused by MCI^5^. Several studies also indicate that aberrant movement patterns during clinical movement tests can be used to identify MCI in patients with CLBP^5, 6, 8–10^.

Lumbar multifidus muscle (LM) is responsible for static and dynamic stability of the lumbar spine^11–14^. Alteration of the LM can cause an inability to control the lumbar spine resulting in MCI. Electromyography studies reveal decreased bilateral LM activity during functional tasks^12, 13^. One study investigated LM activation using ultrasound imaging in patients with CLBP suspected to have underlying MCI^14^. The researchers found lower LM activation in patients with CLBP during remission compared with individuals without LBP^14^. Although reduced LM activation can be resulted from several factors including muscle atrophy, fatty infiltration, or slow-to-fast muscle transition^13^, recent studies demonstrated that LM activation deficit may be caused by reduced neural drive from the primary motor cortex (M1)^15–18^.

Changes in cortical excitability (motor evoked potential, cortical topography, etc.) in patients with CLBP were supported by several studies^15–18^. These changes are also associated with altered movement patterns^15^. Based on the existing evidence, it could be speculated that insufficient LM activation causing MCI should result from changes in cortical excitability (reduced neural drive from the M1)^17, 18^. Accordingly, restoration of LM cortical excitability should increase the neural drive to the LM, thereby increasing LM activation.

Anodal transcranial direct current stimulation (a-tDCS) is a non-invasive neuromodulation technique that can be used to enhance cortical excitability^19–24^. The application of a-tDCS to the M1 area showed a favorable result in cortical excitability and pain^22, 23, 25^. The a-tDCS induces the glutamatergic synapses which in turn enhances the calcium ion influx via N-methyl-D-aspartate (NMDA) receptors^19, 24^. Calcium ion influx has the ability to induce long term potentiation (LTP) which is necessary for neural plasticity^24^. In addition, gamma-aminobutyric acid (GABA) activity can cause the neuron less likely to fire an action potential^19^. By applying a-tDCS, glutamatergic synapses would be activated causing reduced GABA activity, thereby increasing cortical excitability^19^.

Enhanced cortical excitability using a-tDCS should lead to increase in neural drive to the LM. However, this proposed mechanism has not been systematically investigated. In addition, some studies found changes in cortical excitability^15–18^, while others found LM activation deficits in patients with CLBP^12–14^. However, no study has investigated the relationship between cortical excitability and LM activation.

Therefore, this study aimed to investigate the effects of a single session of a-tDCS on cortical excitability and LM activation in patients with CLBP (MCI subgroup) during remission. We also explored the relationship between cortical excitability and LM activation. We hypothesized that a-tDCS would enhance LM cortical excitability, thereby increasing LM activation. We also expected that cortical excitability would be correlated with LM activation in patients with CLBP.

## Methods

### Study design

The study employed a quasi-experimental design to investigate the effect of single session a-tDCS on cortical excitability and LM activation in patients with CLBP (MCI subgroup) during remission.

### Participants

This study used a convenience sampling recruited from university physical therapy clinic and surrounding communities. The inclusion criteria for individuals with CLBP (CLBP) were age between 18 and 40 years, having at least three recurrent episodes for more than three months that interfered with activities of daily living, but currently pain-free, and having more than two positive results of six movement control battery tests to identify MCI including (1) waiter’s bow, (2) standing with posterior pelvic tilt, (3) single leg stance, (4) sitting with knee extension, (5) quadruped rocking forward/backward, and (6) prone with knee flexion^5^. Studies demonstrated people who had higher frequency of aberrant movement patterns during clinical movement tests were likely to have MCI which might be responsible for persistence/recurrence of low back symptoms^5, 8, 10^.

We acknowledged that central pain processing can suppress the LM maximum contraction, and a-tDCS could inhibit this central pain process enabling greater LM contraction^13, 23, 25^. We aimed to prevent the occurrence of this mechanism to investigate our proposed concept. In this study, we investigated patients with CLBP during remission.

The inclusion criteria for individuals without LBP (NoLBP) were no low back pain for the past 6 months and presenting less than two positive movement control battery tests. The exclusion criteria were presence of specific LBP conditions (e.g. degenerative spine, spondylosis, or spinal stenosis), red flags (e.g. infection, tumors, fracture, radicular syndrome, or inflammatory disease), having a diagnosis of neurological, musculoskeletal, or cardiac abnormalities (e.g. scoliosis, myelopathy, atrial fibrillation), receiving motor control training exercises for the past six months, or body mass index greater than 30 kg/m^2^. This study was a part of 6-week intervention study with a pre-specified sample size. Therefore, we did not perform a sample size calculation. However, effect size and the required sample size were calculated for future replications of this study. All participants provided a written informed consent before data collection.

### Instruments

Transcranial magnetic stimulation (TMS; Magstim 200, Magstim Co., UK) was used to stimulate the motor cortex area using a figure-of-eight coil. This system was used to measure the cortical excitability (motor evoked potential; MEP and cortical silent period; CSP) of the LM^18, 22, 26, 27^. One study found moderate to excellent test–retest reliability (ICC_3,1_ = 0.58 to 0.94) to determine cortical excitability^27^. Our pilot data (unpublished work) related to reliability of measurement indicates excellent within-session (ICC_3,k_ = 0.95) and moderate between-session (ICC_2,k_ = 0.67) test–retest reliability. Therefore, TMS was sufficiently reliable to measure cortical excitability.

Rehabilitative ultrasound imaging device (RUSI; model Affiniti 50, Philips, NV, USA) with a broadband curvy linear array (model C6-2) probe was used to measure LM activation at the L4-5 facet joint (2 cm lateral to the lower half of the L4 spinous process). Studies suggest that the relative LM thickness change could represent LM activation^28–30^. Our previous study demonstrated excellent intra-rater reliability (ICC_3,1_ ranging between 0.91 and 0.99), and inter-rater reliability (ICC_2,k_ = 0.95) for LM thickness measurements^30^. Moreover, 95% confidence minimal detectable change was 0.11 cm^30^. Therefore, RUSI was valid and reliable to measure LM activation.

### Procedure

**Figure 1:**
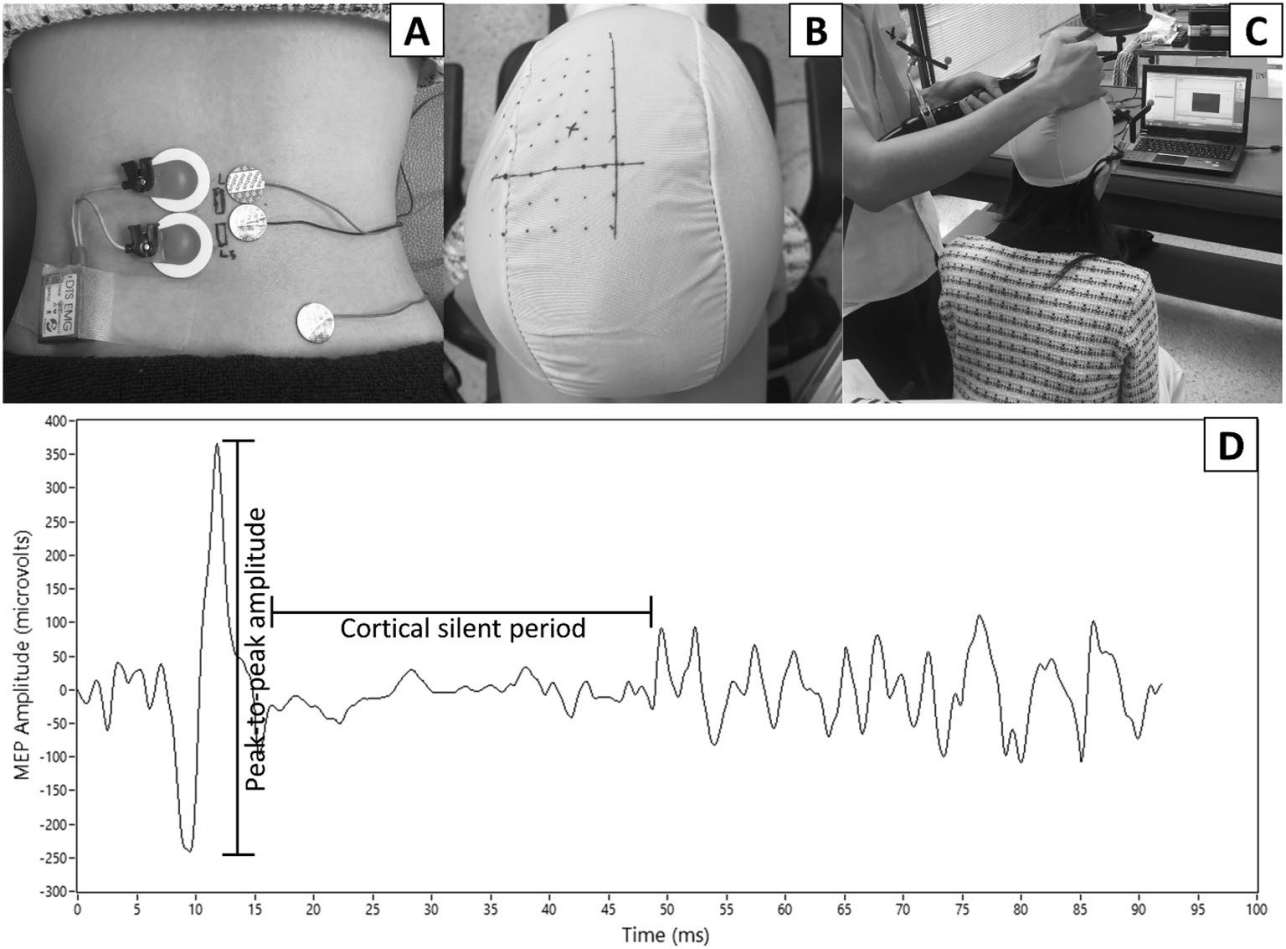
Example of transcranial magnetic stimulation (TMS) protocol to measure cortical excitability of right lumbar multifidus muscle including TMS electrode (**A**, right) and electromyography (**A**, left) placements, pre-marked 5X7 cm swimming cap (**B**), and coil placement during stimulation with visual feedback (**C**). Peak-to-peak motor evoked potential (MEP) amplitude and cortical silent period derived from MEP time-series data (**D**).

This human research followed the principles of the Declaration of Helsiki. Informed consent for publication of identifying information/images in an online open-access publication has also been obtained. The funders played no role in the design, conduct, or reporting of this study. Participants underwent the screening process along with TMS screening questions for eligibility. Eligible participants were asked to fill out demographic data forms. Then, a researcher identified bilateral LM (1 cm lateral to the spinous process of L5)^23^ and prepared the skin using a 70% alcohol swab to reduce skin impedance. EMG electrodes (Telemyo 2400 T G2, Noraxon USA Inc., AZ, USA) were attached on the pain-free side, while TMS electrodes were attached on the painful side based on history of LBP (Fig. 1A). If the history indicated bilateral LBP, a more painful or dominant side was selected. A ground electrode was placed over the posterior superior iliac spine.

The participants were asked to performed two repetitions of three-second maximum voluntary isometric contraction of back extension in the prone with a one-minute rest between repetitions. The averaged MVIC was used to set real-time visual target at 10% MVIC during TMS measurement^15, 18^.

The participants were asked to sit in a TMS chair with their feet flat on the floor and wear a swimming cap marked with a 5 × 7 cm grid relative to the vertex (Fig. 1B). The navigation system along with the 5 × 7 grid was used to consistently position the TMS coil. Then, the participants performed leaning forward to activate the LM until EMG visual feedback reached the 10% MVIC target (Fig. 1C).

A researcher placed a figure-of-eight coil parallel to the scalp site contralateral to the painful side hemisphere in a posterior to anterior direction (Fig. 1C). The researcher used a single-pulse monophasic mode to stimulate each point over the 5 × 7 grid and identified the LM hotspot for each participant^15^. This hotspot was simultaneously registered to the navigation system for repositioning the TMS coil after applying tDCS.

After identifying the hotspot, an active motor threshold was determined using the lowest stimulus intensity to elicit MEP (Fig. 1D) at the hotspot^15, 18^. A MEP amplitude ≥ 200 μV more than 50% of the number of stimuli was needed to activate muscles at 10% MVIC^15, 18^. The intensity of MEP was set at 120% of the active motor threshold stimulus intensity and twenty MEPs were recorded. These data were further used to derive peak-to-peak MEP amplitude (P2P) and cortical silent period (CSP)^15, 22, 26^. CSP was defined as time between the end of the MEP to the point when the LM EMG activity recurred^15, 22, 26^.

**Figure 2:**
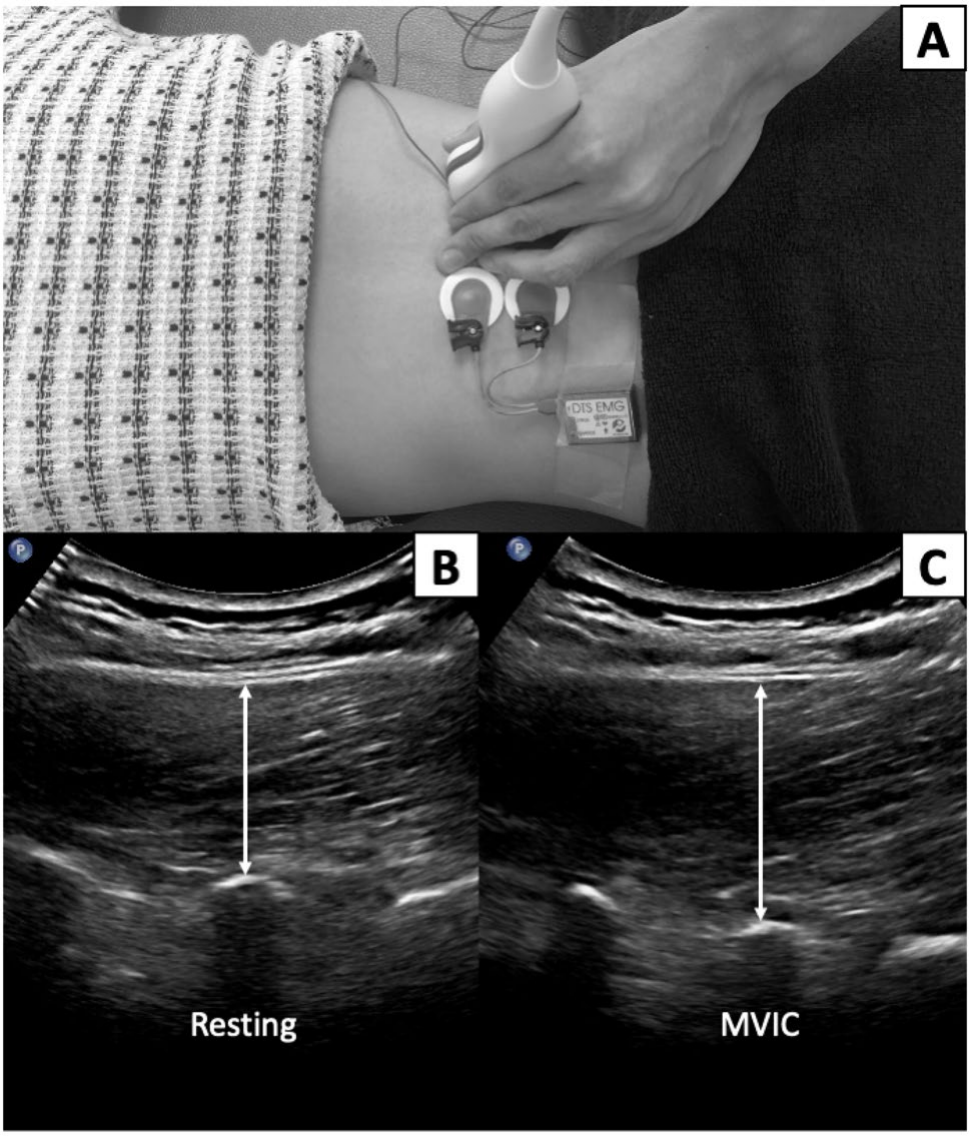
Example of rehabilitative ultrasound imaging protocol to measure right lumbar multifidus muscle (**A**) and muscle thickness during resting (**B**) and maximum voluntary isometric contraction (MVIC; **C**) derived from the distance between tip of L4-5 facet joint and lower border of thoracolumbar fascia.

For RUSI measurement, the participant was asked to lie in the prone position with a towel roll below the abdomen to reduce lumbar lordosis (lumbosacral angle < 10 degrees). The thorax (T3) and pelvis (S2) were securely stabilized to the treatment bed using straps. Then, the researcher placed the RUSI transducer over the painful side of the LM (2 cm lateral to the lower half of the L4 spinous process; Fig. 2A)^14, 30^. Several EMG and MRI studies showed no significant difference between painful and non-painful sides^11–13^. One RUSI study also found no significant difference between painful and non-painful sides in unilateral CLBP, as well as between right and left sides in bilateral CLBP^31^.

The LM thickness was measured during resting (Fig. 2B) twice. Then, the participants were asked to place their hands behind the neck and perform two repetitions of five-second MVIC of back extension with rotation to the opposite side against the external force, while the researcher was recording LM thickness (Fig. 2C)^32^. One-minute rest was provided between repetitions to prevent muscle fatigue. The distance between the tip of the L4-5 facet joint and thoracolumbar fascia was measured for both conditions^14, 30^. Percent LM thickness change from resting was used to represent LM activation. Our previous study indicated excellent intra-rater (ICC_3,1_ = 0.987–0.996) and inter-rater reliability (ICC_2,k_ = 0.978–0.997)^30^. Moreover, 95% confidence minimal detectable change was 0.11 cm^30^.

After baseline collection, only the CLBP group received anodal tDCS (a-tDCS) using 5X7 cm electrodes in which the anodal electrode was placed on the hotspot representing the LM in the M1 area contralateral to the painful side, while the cathodal electrode was attached to the ipsilateral supraorbital area^22, 23^. The a-tDCS was applied to the participants in sitting position. Participants were asked to stay awake during stimulation. The intensity was set at 2 mA with 10-s fade in/out^22, 23^. The participants were stimulated by a-tDCS for 20 min. Then, TMS and RUSI measurements were performed again to obtain post-intervention data.

### Statistical analysis

All statistical analyses were performed using SPSS Software (IBM SPSS Statistics 23 for Windows). Descriptive statistics was used to analyze participant characteristics, and the Shapiro–Wilk test was used to determine normality of the data. We found that our data were normally distributed; therefore, an independent *t* test was used to compare cortical excitability (P2P and CSP) and LM activation between groups, while a paired t-test was used to compare the differences between pre- and post-intervention for the CLBP group. In addition, Pearson’s correlation coefficient was used to examine the association between cortical excitability and LM activation. We separately analyzed correlations for each group because healthy individuals might not have any impairment; therefore, there should not be any correlation between cortical excitability and LM activation. We also performed correlation at post-intervention in the CLBP group to ensure whether we found similar correlation compared with pre-intervention.

### Ethics approval and consent to participate

This study was approved by the Institutional Review Board of Mahidol University (COA No. MU-CIRB 2021/184.0309) and registered in clinical trial (ClinicalTrials.gov ID: NCT05156242).

## Results

Thirteen healthy participants and 38 participants with low back pain were screened for eligibility. Of 13 healthy participants, 2 participants had more than 2 positive movement control battery tests; therefore, 11 participants without LBP were included for this study. For participants with low back pain, 13 participants did not meet movement control screening and 12 participants did not meet the definition of CLBP. Therefore, 13 participants with CLBP were included in this study. No significant differences (*P* > 0.05) were found in participant characteristics between groups, except frequency of positive movement control battery tests in which the CLBP group had significantly greater frequency (*P* < 0.05) than that of the NoLBP group (mean frequency = 0.9 and 3.8, respectively). The participant characteristics are presented in Table 1.

**Table 1:** Participant characteristics.

Parameter	NoLBP (n = 11)	CLBP (n = 13)
Age (years)	25.4 ± 3.0	29.4 ± 6.8
Height (m)	1.6 ± 0.1	1.6 ± 0.1
Weight (kg)	52.7 ± 10.0	59.2 ± 14.6
BMI (kg/m^2^)	20.3 ± 2.0	22.1 ± 3.9
Gender (%female)	8 (72.7)	7 (53.8)
Duration of LBP (year)	N/A	3.8 ± 3.8
Recurrence episodes (within 6 months)	N/A	9.8 ± 7.8
Frequency of positive movement control battery tests	0.9 ± 0.6	3.8 ± 0.8*

*BMI* body mass index, *LBP* low back pain, *No LBP* no low back pain group, *CLBP* chronic low back pain group.

*Significant difference between groups (p < 0.05).

**Figure 3:**
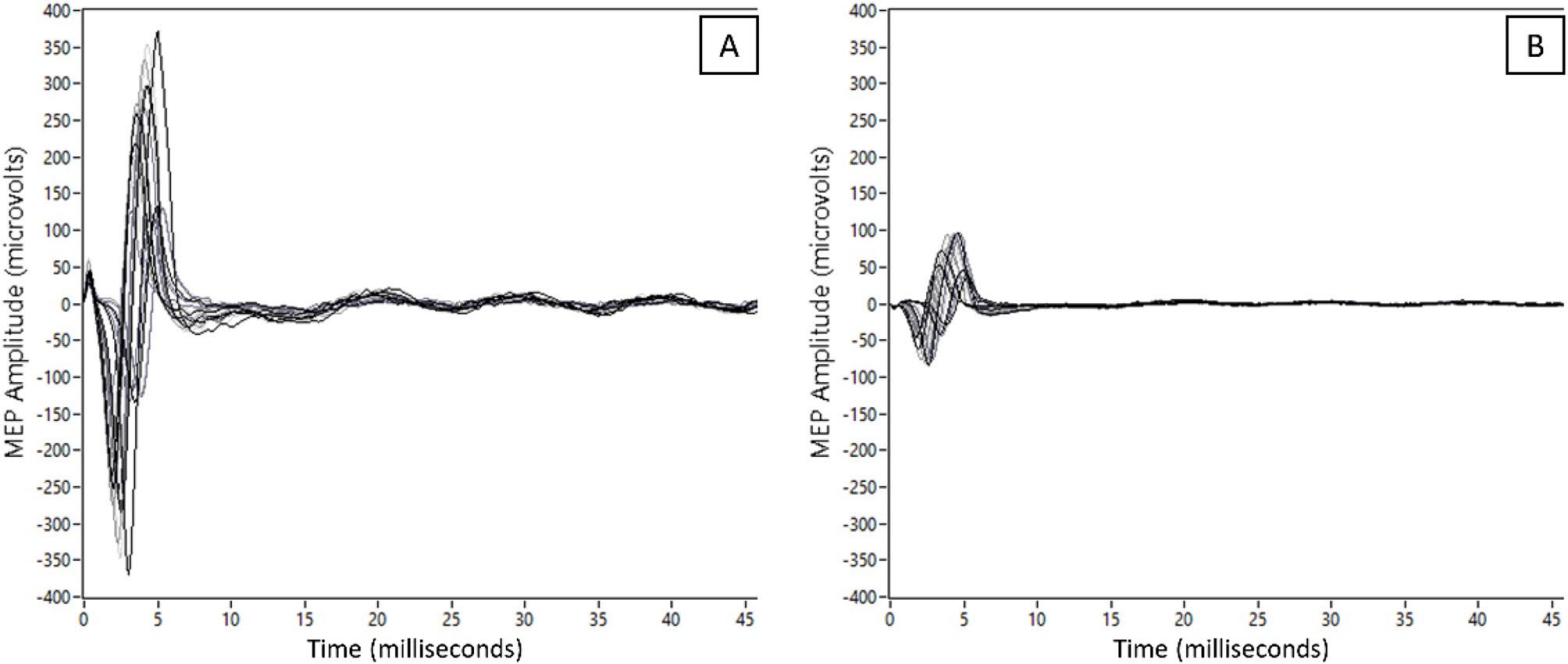
Example of an overlay MEPs from individual without LBP (**A**) and individual with CLBP (**B**).

Figure 3 demonstrates example of MEPs from individual without LBP (A) and individual with CLBP (B). Statistical analysis demonstrated significant difference in P2P between groups. However, no significant differences were found in P2P, CSP, and LM activation between pre- and post-intervention. Between groups and within-group comparisons are presented in Table 2. In addition, a trend indicating moderate positive correlation was observed between P2P and LM activation (r = 0.55, *P* = 0.05) in CLBP group pre-intervention (Table 3), while a strong positive correlation (r = 0.72, *P* = 0.006) was found post-intervention. No significant correlations (*P* > 0.05) were found between CSP and LM activation in the CLBP group. The NoLBP group did not show any significant correlation (*P* > 0.05) between cortical excitability and LM activation. In addition, no participants reported serious adverse effects of a-tDCS and only one participant reported an itchy sensation in the forehead during a-tDCS application. However, it did not last more than one day.

**Table 2:** Pre- and post-comparison of peak-to-peak motor evoked potential amplitude (P2P), cortical silent period (CSP), and lumbar multifidus muscle (LM) activation.

Outcome	NoLBP	CLBP	
		Pre	Post
P2P (microvolts)	350.0 ± 185.3	206.8 ± 127.6*	195.2 ± 75.0
CSP (milliseconds)	53.3 ± 75.0	109.4 ± 100.4	75.2 ± 149.6
LM activation (%)	39.4 ± 13.6	33.7 ± 17.5	36.2 ± 15.5

*No LBP* no low back pain group, *CLBP* chronic low back pain group.

*Significant difference between groups (p < 0.05).

**Table 3:** Correlation between cortical excitability and lumbar multifidus muscle (LM) activation.

Parameters	NoLBP		CLBP			
			Pre		Post	
	r	p-value	r	p-value	r	p-value
P2P and LM activation	− 0.21	0.54	0.55	0.05	0.72	< 0.05*
CSP and LM activation	− 0.28	0.41	− 0.11	0.72	− 0.11	0.74

*No LBP* no low back pain group, *CLBP* chronic low back pain group.

*Significant correlation (p < 0.05).

## Discussion

Our study was designed to investigate the immediate effects of a-tDCS on cortical excitability and LM activation and explore the relationship between cortical excitability and LM activation in participants with CLBP during remission. We first compared whether our participants with CLBP actually had changes in cortical excitability and LM activation. P2P finding supports that those participants with CLBP had decreased MEP amplitude. Although we found trends in which participants with CLBP had shorter CSP and lower LM activation, these findings did not yield statistical significance.

Several studies reported changes in cortical excitability in patients with CLBP^15–18^. Researchers suggest that these changes can be caused by reflex inhibition response to pain/injury^16, 33^. This reflex inhibition is reflected as muscle activation and movement pattern alterations that may aim to minimize the load on injured structures^16, 33^. In addition, the repetition of adapted muscle activation and movements over time results in neuroplastic changes that can be represented as altered cortical excitability within the motor cortex^15–18^.

The non-significant increase in MEP amplitude after a-tDCS intervention did not support our hypothesis in which we expected that tDCS can modulate cortical excitability. This finding is inconsistent with the study reporting the immediate effect of a-tDCS on cortical excitability^22^. However, those studies applied a-tDCS in healthy individuals which could cause different responses compared with those of patients with CLBP^22^.

Another potential explanation is the reversed effect of a-tDCS. Theoretically, a-tDCS should induce increased MEP amplitude by depolarization of the resting membrane potential causing the long-term potentiation (LTP)-like plasticity effect^19, 24^. However, a-tDCS might induce long-term depression (LTD)-like plasticity resulting in inhibiting the excitability^34, 35^. Another mechanism is that a-tDCS can induce increase in cortical excitability by opening the Ca^+^ influx^19, 24^. However, if an overwhelming Ca^+^ occurs in the voltage channel, the counter regulation mechanism will take place in this process which in turn blocks the N-methyl-D-aspartate (NMDA) receptor and could be eventually result in decreased cortical excitability^34^.

Although between-participant variability with different underlying neurophysiological mechanisms could be responsible for non-significant changes in cortical excitability in other studies^36, 37^, our study specifically included participants with the same underlying MCI. Therefore, the effect of variability in our participants on non-significant changes is unlikely.

The intra-subject differences under their neurophysiological mechanism such as emotional changes, stress, fatigue, variation over time and hormonal changes occurred in our study^38^. Although we controlled the participant characteristics and time of the day for the investigation, we did not control their neurophysiological conditions that might have affected the response to a-tDCS^38^.

Significant difference in MEP amplitude between healthy individuals and participants with CLBP indicated participants with CLBP actually experienced a change in cortical excitability. This would suggest that our one session of a-tDCS at 2 mA for 20 min might not have been sufficient to increase cortical excitability. Therefore, further study is needed to determine appropriate doses and explore more possible outcomes by considering and controlling possible neurophysiological mechanisms using a true experimental study design.

No significant change in CSP post-intervention also did not support our hypothesis. This could have been due to cortical inhibition caused by GABA activity^24, 39^. The a-tDCS should activate NMDA receptors and induce calcium-dependent plasticity; however, excessive Ca^+^ might trigger GABA activity to counter regulate by blocking the NMDA receptor resulting in unchanged CSP^24, 39^. Another explanation might be our participants did not experience CSP change compared with their NoLBP counterpart. In addition, several studies found high variability in CSP and suggested that CSP might be unreliable measurement to investigate neurophysiological response in participants with CLBP^37, 38^.

Our finding did not show increase in LM activation after a-tDCS intervention as we expected. No study has investigated the effect of a-tDCS on LM activation; therefore, we were unable to compare and contrast with other studies. However, studies commonly used a-tDCS to reduce pain^23, 25^. The presence of pain can cause reflex inhibition to LM leading to inability to perform maximum contraction^14, 30^. One of proposed effects of a-tDCS is to reduce pain which could prevent reflex inhibition, thereby indirectly increasing LM activation. Our study recruited participants with CLBP during remission, and non-significant difference in LM activation between groups suggests no LM activation deficit in our participants with CLBP. Therefore, this mechanism might not have taken place in our study.

The correlation between P2P and LM activation in participants with CLBP partially supports our hypothesis in which decreased MEP amplitude would result in LM activation deficit. Some studies demonstrated changes in cortical excitability^15–18^, while others found LM activation deficits in patients with CLBP^12–14^. Those researchers assumed a relationship existed between cortical excitability and LM activation. Our moderate (pre-intervention) and strong (post-intervention) positive correlations did support the existence of this relationship. These correlations suggest that cortical excitability may play a critical role in LM activation; therefore, intervention should include a neuromodulator to enhance cortical excitability. One study reported no relationship between changes in cortical excitability and LM activation in patients with CLBP^15^. The discrepancy between studies could be because our study included participants with CLBP with underlying MCI as a homogeneous group, while their study might have included CLBP with different underlying mechanisms.

Although our study did not show improved cortical excitability and LM activation after a-tDCS intervention, we found moderate to strong positive correlations between MEP amplitude and LM activation. These findings at least suggest the involvement of the cortical level in CLBP. Therefore, clinicians should include interventions involving neuromodulation to treat patients with CLBP with underlying movement control impairment.

## Limitations

Limitations were encountered in this study. Our participants with CLBP were specific to pain-free condition, age between 20 and 40 years, and presenting at least three positive movement control tests. These characteristics would limit generalization in a CLBP population. We were able to recruit only a small sample size and our study used a quasi-experimental design due to difficulty in recruiting participants with specific criteria. However, we used healthy individuals as reference to compare at baseline to ensure the existence of changes in cortical excitability and LM activation in participants with CLBP. Replication of our study employing a larger sample and randomized controlled trial is needed. We performed post-hoc power analysis for our correlation findings (r = 0.55 and 0.72) with 95% confidence and sample size of 13 (CLBP group). We found achieved power of 0.58 and 0.92, respectively. In addition, a minimum sample size of 21 and 10 participants would be required to replicate our study using these correlation coefficients, 80% power and confidence level of 0.05. Decreased LM activation could be resulted from other factors, such as muscle atrophy, fatty infiltration, or slow-to-fast muscle transition. Therefore, future study should take these potential confounding factors into consideration. We used single-pulse TMS to investigate excitability of motor cortex corresponding to painful side only. This would limit the investigation of intra-cortical connections within a specific brain region and inter-cortical connections between different brain regions. Therefore, future study may use paired-pulse paradigm to provide a more comprehensive understanding of excitatory and inhibitory mechanisms.

## Data Availability

The datasets used and/or analyzed during this study would be available from corresponding author upon reasonable request.
